# Preeclampsia and associated factors among pregnant women attending antenatal care in Dessie referral hospital, Northeast Ethiopia: a hospital-based study

**DOI:** 10.1186/s12884-015-0502-7

**Published:** 2015-03-29

**Authors:** Gizachew Assefa Tessema, Abebe Tekeste, Tadesse Awoke Ayele

**Affiliations:** Department of Reproductive Health, Institute of Public Health, University of Gondar, Gondar, Ethiopia; Meseret Desta Specialized Higher Clinic, Dessie, Ethiopia; Department of Epidemiology and Biostatistics, Institute of Public Health, University of Gondar, Gondar, Ethiopia

**Keywords:** Prevalence, Preeclampsia, Ethiopia

## Abstract

**Background:**

Preeclampsia is one of the leading causes of maternal mortality in Ethiopia. It has been increasing and linked to multiple factors, making prevention of the disease a continuous challenge. Yet few studies have been conducted in Ethiopia. This study aimed to assess the prevalence and factors associated with preeclampsia among pregnant women attending antenatal care in Dessie referral hospital, Northeast Ethiopia.

**Methods:**

A hospital-based cross-sectional study was conducted in Dessie referral hospital between August and September 2013. All pregnant women who had antenatal visit at Dessie referral hospital were included for the study. A total of 490 pregnant women were enrolled in the study. Pretested and structured questionnaire via face-to-face interview technique was used for data collection. The data were entered in to EPI info version 3.5.3 statistical software and exported to SPSS version 20 statistical package for further analysis. Descriptive statistics were used to explore the data in relation to relevant variables. Binary logistic regression analysis was employed. Odds ratio with 95% confidence intervals (CI) was computed to identify factors associated with Preeclampsia.

**Results:**

The prevalence of preeclampsia among pregnant women in Dessie referral hospital was found to be 8.4%. Women having family history of hypertension [Adjusted Odds Ratio (AOR) = 7.19 (95% CI 3.24–15.2)], chronic hypertension [AOR = 4.3 (95% CI 1.33–13.9)], age ≥35 years [AOR = 4.5 (95% CI 1.56–12.8)], family history of diabetes mellitus [AOR = 2.4 (95% CI 1.09–5.6)] and being unmarried [AOR = 3.03 (95% CI 1.12 – 8.2)] were found to be associated with preeclampsia.

**Conclusions:**

The prevalence of preeclampsia in this hospital was lower that other studies. Having personal or family history of hypertension, older age, and family history of diabetic mellitus were factors associated with preeclampsia. Encouraging pregnant women to have health seeking behavior during pregnancy would provide a chance to diagnose preeclampsia as early as possible.

## Background

Approximately 289,000 women died globally from pregnancy-related causes in 2013. Of which, 99% of deaths occur in developing nations. Sub-Saharan Africa accounts for about 56% of all maternal deaths. A woman’s lifetime risk of dying from pregnancy-related complications in developing countries is 14 times higher than in developed countries [1]. According to 2011 Ethiopia Demographic and Health Survey (EDHS) report, an estimated 676 women per 100,000 live births were dying of pregnancy and related causes [2]. Sixty to eighty percent of all maternal deaths are due to five major complications-namely, postpartum hemorrhage, puerperal sepsis, Hypertension disorder of pregnancy, unsafe abortion and obstructed labor [3].

Hypertensive Disorder of Pregnancy (HDP) is one of the leading causes of maternal mortality and morbidity amongst pregnant women in the world [4]. The World Health Organization (WHO) estimates of maternal death due to HDP were 25.7% in Latin-American and Caribbean, and 9.1% in Asian and African countries [5,6]. A study conducted in Ghana revealed that pregnancy induced hypertension has contributed for 8.9% maternal mortality [7].

WHO estimated the incidence of preeclampsia to be seven times higher in developing countries than developed countries [4]. A study done in Western Shoa found that 12.3% maternal mortality occurred from hypertension disorder of pregnancy [8]. The Ethiopian National Emergency Obstetric and Newborn Care (EMONC) showed that preeclampsia contributed for the complication of approximately 1% of all deliveries and 5% of all pregnancies. Moreover, 16% of direct maternal mortality and 10% of all maternal mortality (direct and in direct) was due to preeclampsia/eclampsia [9]. A maternal mortality trend analysis showed an increasing trend of preeclampsia in Ethiopia [10]. Globally, it is the causes of nearly twelve percent of direct maternal deaths [11]. Hypertensive disorder of pregnancy has remained a significant global public health threat in both developed and developing countries that contribute to maternal and perinatal morbidity and mortality. However, there are few studies in Ethiopia [9,12], these have different objectives and study populations. Hence, this study can assess the prevalence and associated factors of preeclampsia among pregnant women in Dessie referral hospital, Northeast Ethiopia.

## Methods

### Study design, population, and setup

A hospital-based cross-sectional study was conducted between August and September 2013 in Dessie referral hospital, Northeast Ethiopia. Dessie is located 401 kilometers away from the capital, Addis Ababa. The hospital has two hundred beds and serving for about seven million people living in the catchment areas. It also provides comprehensive obstetric care.

The study populations were pregnant women who attended antenatal care in the hospital during the study period. Those pregnant women with a gestational age of 20 weeks or greater were included in the study. Pregnant women’s gestational age was measured based on women recall of the last menstrual period (LMP). Ultrasound estimation for gestational age was also considered when women fail to remember the LMP but underwent for ultrasound evaluation. When the gestational age estimation was not possible in both cases, pregnant women were excluded from the study to prevent misclassification. Sample size was estimated using single proportion formula using the following assumptions: Proportion of preeclampsia (P) 7.6% [12], 95% CI, 2.5% margin of error, a non-response rate of 10%, the total sample size was calculated to be 475. Women who visited the ANC clinic at hospital from August to September 2013 were included.

### Operational definitions

*Preeclampsia* is defined as gestational hypertension [systolic blood pressure (SBP) ≥140 mmHg and/or diastolic blood pressure (DBP) ≥90 mmHg] after 20 weeks of gestation plus the presence of proteinuria. *Proteinuria* is assessed using urine dipstick method. Those women having a protein level of 1+ classified as having proteinuria.

### Data collection tools and procedures

Data were collected by face to face interview technique using structured and pretested questionnaire. The questionnaire was first prepared in English then translated to local language (Amharic) and back to English to maintain conceptual consistency. Four midwives and one supervisor were involved in the data collection process. Medical records were also reviewed for some clinical and laboratory results including proteinuria. Data collectors and supervisor were taken two days training on interviewing technique, the objective of the study, and the different sections of the questionnaire. The questionnaire was pretested at Dessie Health Center. The participants were allowed to take rest for ten minutes before the blood pressure had been measured. Blood pressure readings were taken while the woman was seated in the upright position using a mercury sphygmomanometer apparatus which covers two-thirds of the upper arm. The measurement was taken from participant’s right hand. The cuff was inflated at a rate of 2–3 mmHg per second. Systolic blood pressure (SBP) was taken up on hearing the first sound, and diastolic blood pressure (DBP) was taken up on 4^th^ (muffled) Korotkoff sound. Those pregnant women with abnormal findings were checked again and again and then have undergone for another BP measurement after 4–6 hours in order to confirm the diagnosis. For the sake of assuring whether the BP apparatus was functioning correctly, the data collector checked it by measuring the blood pressure of other data collector. However, when a pregnant woman found to have severe preeclampsia (BP of 160/110 mmHg), she was sent for immediate re-checkup and medical advice. Data regarding proteinuria and other clinical data were accessed from the women’s medical records. Proteinuria was assessed using urine dipstick method and was part of the routine investigation for all pregnant women.

### Data processing and analysis

The filled questionnaires were checked for completeness, cleaned manually and entered in to EPI INFO version 3.5.3 statistical software and then transferred to SPSS version 20 statistical package for further analyses. Descriptive statistics were used to explore the data in relation to relevant variable. Binary logistic regression was used to assess the association between the dependent variable and independent variables. Then variables with P-value less than or equal to 0.2 were fitted to multiple logistic regression. The predictive ability of the model was tested with Hosmer-Lemeshow goodness-of-fit test. Finally, variables with P- value less than 0.05 were considered as factors associated with preeclampsia.

### Data quality control

Data collectors and supervisor has been given training on how to approach the participants and perform measurements. The performance of the instruments was checked and measurement tools monitoring were done. Participants were asked to remove tight outer-wearing and shoes. Blood pressure measurement was taken by one nurse so as to avoid the inter-observer bias. The supervisor and the principal investigator checked questionnaires on daily basis for inconsistencies and omissions.

### Ethical considerations

Ethical clearance was obtained from the Ethical Review Board of Institute of Public Health, College of Medicine and Health Sciences, University of Gondar. Communication with the hospital administrators were made through formal letter obtained from the Institute of Public Health. After the purpose of the study has been informed, written consent was obtained from each study participants. Confidentiality was maintained by making the data collection procedure anonymous. Participation was on voluntary basis. Women who have been found preeclamptic were advised to follow antenatal care strictly.

## Results

### Socio-demographic characteristics of the study participants

A total of four hundred ninety pregnant women were enrolled in the study. The mean age of the participants was 27 years (SD = 4.6). Most (91.4%) were married and 263 (53.7%) participants were Muslims in religion. Two hundred seventy six (56%) of them attended secondary school or above. Majority (88.6%) of participants identified Amhara as their ethnic origin and two hundred sixty four (53.9) were housewives (Table 1).

**Table 1:** **Socio-demographic characteristics of women attending antenatal care at Dessie referral hospital, 2013 (n = 490)**

**Variables**	**Frequency**	**Percentage**
**Age (in years)**		
≤24	141	28.8
25 – 29	209	42.7
30 – 34	97	19.8
≥35	43	8.8
**Marital status**		
Currently married	448	91.4
Currently unmarried	42	8.6
**Religion**		
Orthodox	206	42
Muslim	263	53.7
Protestant	21	4.3
**Ethnicity**		
Amhara	434	88.6
Oromo	35	7.1
Others*	21	4.3
**Educational status**		
Unable to read and write	52	10.6
Able to read and write	77	15.7
Primary education	85	17.3
Secondary education	167	34.1
Tertiary level	109	22.2
**Occupation**		
Government/private employee	111	22.7
House wife	264	53.9
Merchant	62	12.7
Other**	53	10.8

*others = Afar and Tigray **others = students, laborers and farmer.

### Clinical characteristics of the respondents

Seventeen (3.5%) of them had systolic blood pressure of 140 mmHg or more. In addition, 24 (4.9%) of women had diastolic BP of 90 mm Hg or greater Four hundred and nineteen (85.5%) had negative urine protein test. Most (91%) had normal blood pressure and 18 (3.7%) of them had chronic hypertension. The prevalence of preeclampsia was found to be 41 (8.4%) [95% CI: 6.2–11.8)].

### Reproductive and obstetric characteristics of the respondents

From the total participants, 374 (76.3%) started menstruating at the age of 13–15 years. Concerning the first age of pregnancy, 6 (1.2%) became pregnant at 35 years or above. One hundred and forty one (28.8%) of the respondents were pregnant for the first time. About 16 (3.3%) of pregnant women has history of twin pregnancies and 15 (3.1%) of them has history of infertility (Table 2).

**Table 2:** **Reproductive and obstetric characteristics of women attending antenatal at Dessie referral hospital, Ethiopia, 2013 (N = 490)**

**Variables**	**Frequency**	**Percentage**
**Age at menarche (in years)**		
≤12	58	11.8
13 – 15	374	76.3
≥16	58	11.8
**Age at first pregnancy (in years)**		
<35	446	98.8
≥35	6	1.2
**Gravidity**		
1	141	28.8
≥2	349	67.8
Change paternity after previous pregnancy (n = 349)*		
Yes	54	15.5
No	295	84.5
**History of infertility**		
Yes	15	3.1
No	475	96.9
**Parity**		
Nullipara	168	34.3
1 delivery	167	34.1
≥2 deliveries	155	31.6
**Multiplicity of pregnancy**		
Singleton	474	96.7
Twin	16	3.3

* = had no previous pregnancy.

### Behavioral and family history related characteristics

In this study eight (1.6%) women were taking tobacco while 21 (4.3%) of them were drinking alcohol. Four hundred and one (81.8%) had no family history of hypertension, but 56 (11.6%) had family history of diabetes mellitus (Table 3).

**Table 3:** **Behavioral characteristics and family history of women attending antenatal care at Dessie referral Hospital, 2013 (N = 490)**

**Variables**	**Frequency**	**Percentage**
**Tobacco use**		
Yes	8	1.6
No	482	98.4
**Alcohol use**		
Yes	21	4.3
No	469	95.7
**Family history of HTN**		
Yes	89	18.2
No	401	81.8
**Family history of DM**		
Yes	58	11.4
No	432	88.6

### Factors associated with preeclampsia

The association between socio-demographic, obstetrical, family history of the women, and preeclampsia were assessed. In the bivariate analysis; maternal age, marital status, education, gravidity, family history of hypertension and family history of diabetes mellitus became significant at 0.2 level of significant. However, maternal age, family history of hypertension, having chronic hypertension, family history of diabetes mellitus and marital status were remained significantly and independently associated with preeclampsia in the multivariate analysis.

The development of preeclampsia was increased with pregnancies in older ages. Those women in age category 35 and above had 4.5 times higher odds of developing preeclampsia than those aged 25–29 years [AOR 4.5, 95% CI 1.56 –12.8)]. Likewise, those pregnant women aged 30–34 years had about 3.3 times higher odds developing preeclampsia than those women between 25–29 years of age [AOR 3.26, 95% CI (1.35–7.8)].

Those women with family history of hypertension had about 7.2 times higher odds of developing preeclampsia than women who haven’t [AOR 7.19, 95% CI 3.4–15.2)] . Those women with family history of diabetes mellitus had 2.4 times higher odds of developing preeclampsia as compared to those with no family history. [AOR 2.4, 95% CI 1.09 – 5.6)].

Those currently unmarried pregnant women had about 3 times higher odds of developing preeclampsia than those married counterparts [AOR 3.03. 95% CI 1.12–8.2)] (Table 4).

**Table 4:** **Bivariate and multivariate logistic regression analysis of factors associated with preeclampsia among pregnant women in Dessie referral hospital, 2013 (N = 490)**

**Variables**	**Preeclampsia**		**OR (95% CI)**	
	**Yes**	**No**	**COR (95% CI)**	**AOR (95% CI)**
**Age (in years)**				
≤24	3	138	0.36 (0.09 – 1.29)	0.46 (0.12 – 1.76)
25 – 29	12	197	1.00	1.00
30 – 34	16	81	3.24 (1.47 – 7.16)*	3.26 (1.35 – 7.8)*
≥35	10	33	4.97 (1.99–12.4)*	4.5 (1.56 – 12.8)*
**Marital status**				
Currently unmarried	9	33	3.55 (1.56 - 8.05)*	3.03 (1.12- 8.2)*
Currently married	32	416	1.00	1.00
**Education status**				
Unable to read/write	4	48	1.73 (0.46 – 6.74)	
Able to read/write	12	65	3.84 (1.29 – 11.4)	
Primary education	9	76	2.46 (0.79 – 7.64)	
Secondary education	11	156	1.47 (0.49 – 4.34)	
Tertiary level	104	5	1	
**Gravidity**				
1	8	133	1.74 (0.78 - 3.86)	
>1	33	316	1.00	
**Chronic HTN**				
Yes	6	12	6.2 (2.2 - 17.6)*	4.3 (1.33 – 13.9)*
No	35	437	1.00	1.00
**Family history HTN**				
Yes	25	64	9.39 (4.76 - 18.57)*	7.19 (3.4 - 15.2)*
No	16	385	1.00	1.00
**Family history DM**				
Yes	14	44	4.77 (2.33 - 9.77)*	2.4 (1.09 – 5.6)*
No	27	405	1.00	1.00

*P-value < 0.05.

## Discussion

Preeclampsia is pregnancy induced hypertension with significant proteinuria. It is one of the major causes of maternal mortality worldwide. The current study revealed that the prevalence of preeclampsia was 8.4% [95% CI: 6.2–11.8)]. The finding was similar with a study done at Jimma University Hospital, Ethiopia (7.6%) [12], and Iran (9.5%) [13], but higher than the finding from developed countries (1.4%–5.0%) [14]. However, it is lower than the findings of studies conducted in Birmingham (11.9%) [15], Nigeria (16%) [16], and Northern Finland (13.9) [17]. The difference between the present finding and other studies might be due to the geographic differences and study design.

The association of maternal age and development of preeclampsia was declared in studies conducted at Finland [18] and Iran [19]. With this regard, the current study showed the presence of higher odds of developing preeclampsia in older women. Those pregnant women who were 35 or above had four times more odds of develop preeclampsia than those 25–29 years old. Likewise, those women aged 30–34 years were about three times more odds of developing preeclampsia than those 25–29 years old. This could be explained as woman gets older, she is more likely to have cardiovascular problems. This would particularly happen due to the gradual loss of compliance of the cardiovascular vessels that is mainly associated with ageing of uterine blood vessels and arterial stiffness. In addition, when woman gets older, the haemodynamic adaptation during pregnancy become more difficult [20].

Those women with family history of hypertension had about seven times greater odds of developing preeclampsia compared those who haven’t. This finding is in line with studies conducted in Brazil [21], Sudan [22], Pakistan [23], and Uganda [24]. This might have occurred due to genetic factors that contribute to the physiologic predisposition of preeclampsia.

Marital status had statistically significant association with the development of preeclampsia. Being currently unmarried were about three times more likely to develop preeclampsia than those who were married. The finding was in line with the study conducted in USA [25]. The reason might be explained by the possibility of low preconception period seminal fluid exposure among unmarried women. This seminal familiarity would obtain from frequent insemination by the same partner for long period of time and hence could lead to the development of preeclampsia. [26,27]. On the other hand, it might also be explained by stress that arisen from delivering a child without father brought less social acceptability and economical support.

Those pregnant women with family history of diabetes mellitus were about two times more likely to develop preeclampsia. The report in the present study was in line with the research done in Thailand [28]. Genetic factors might be responsible to predisposing women to an increase risk of preeclampsia.

Preexisting hypertension had statistically significant association with preeclampsia. Being women with preexisting hypertension were about four times more likely to develop preeclampsia. This finding was in concordance with the researches done in France [29] and Nigeria [16].

However, unlike other literature [28], tobacco smoking is not significant in this study. This might be due to the presence of few numbers of women in this categories.

The current study has some limitations. First, it may have recall bias regarding some factors such as time of menarche. Secondly, biochemical tests had not been done for exclusion of women with other chronic diseases. Thirdly, the cross-sectional nature of the study didn’t allow us to confirm whether non-preeclamptic women remained negative until delivery or not. This study would not also be generalized for population level.

## Conclusions

The prevalence of preeclampsia in this hospital was lower with other similar studies. Having personal or family history of hypertension, older maternal age, and family history of diabetic mellitus were factors associated with Preeclampsia. Encouraging pregnant women’s health seeking behavior would provide a chance to diagnose preeclampsia as early as possible.
